# Sustainable Nonenantioselective Production and Stereochemical Characterization of the Lignin‐Derived Chiral Building Block 3‐Carboxymuconolactone

**DOI:** 10.1002/open.202500453

**Published:** 2026-01-05

**Authors:** Yuzo Suzuki, Takuma Araki, Masaya Fujita, Naofumi Kamimura, Eiji Masai, Tsuyoshi Michinobu, Yuichiro Otsuka, Shojiro Hishiyama, Masaya Nakamura

**Affiliations:** ^1^ Department of Forest Resource Chemistry Forestry and Forest Products Research Institute Tsukuba Japan; ^2^ Department of Materials Science and Bioengineering Nagaoka University of Technology Nagaoka Japan; ^3^ Department of Materials Science and Engineering Institute of Science Tokyo Meguro‐ku Japan

**Keywords:** 3‐carboxymuconolactone, biomass, chiral building block, lignin, renewable resources

## Abstract

To promote the comprehensive utilization of renewable lignocellulosicbiomass, a practical technology for the nonenantioselective production of 3‐carboxymuconolactone (3CML), a lignin‐derived chiral building block, is presented. Although an engineered *Pseudomonas putida* strain with plasmids containing bacterial and fungal genes was previously used to convert lignin‐derived aromatic compounds into optically pure 4*S*‐3CML, using the enantiomeric pair 4*S*‐3CML and 4*R*‐3CML as polymer building blocks in appropriate blending ratios can be expected to afford novel materials such as polylactic acid with tunable physical properties for targeted applications. Therefore, in this study, *P. putida* was engineered to convert vanillic acid, the major aromatic compound derived from lignin, into 3‐carboxy‐*cis*,*cis*‐muconate, which was then chemically converted into racemic 3CML under acidic conditions. Using a chiral high performance liquid chromatography–circulardichroism system, racemic 3CML was stereochemically characterized on the basis of the enantiomers. A one‐pot process for the production of racemic 3CML was established by combining fed‐batch fermentation with subsequent acidic treatment using a jar fermenter, affording 6.6 g/L 4*S*‐3CML and 7.2 g/L 4*R*‐3CML in a high yield of 93.1%. The developed process can be consistently performed at 28°C without requiring pressure or metal reagents and allows using a reduced volume of solvent, offering clear advantages for industrial applications.

## Graphical abstract

The first stereochemical characterization and sustainable nonenantioselective production of 3‐carboxymuconolactone (3CML), a lignin‐derived chiral building block, are presented. The developed process consistently proceeds at 28°C without requiring pressure or metal reagents and allows reducing the amount of solvent, offering clear advantages for industrial applications.

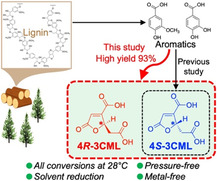

## 1 Introduction

To realize the transition from fossil‐based resources such as petroleum to renewable biomass resources, the development of sustainable technologies to produce fine chemicals and functional materials from biomass resources is essential. In this context, polylactic acid stands out as one of the most representative and widely used industrially bio‐based polymers. Polylactic acid is produced from lactic acid, a chiral compound that exists as a pair of enantiomers (L‐ and D‐lactic acid). Controlling the blending ratios of the enantiomers allows generating polylactic acid with tunable glass transition and melting temperatures for specific applications [1]. In addition, the mixture of approximately equal amounts of L‐ and D‐lactic acid affords poly(D, L‐lactic acid), which is preferred for drug delivery applications over single‐enantiomer polymers owing to its faster biodegradation rate [2]. Furthermore, blending enantiomerically pure poly(L‐lactic acid) and poly(D‐lactic acid) results in a stable stereocomplex with superior characteristics to those of single‐enantiomer polymers, including mechanical strength, thermochemical properties, and hydrolysis resistance [3]. Thus, diverse polylactic acids suitable for specific functions can be manufactured by exploiting the inherent advantages of the chiral building blocks.

The industrial production of lactic acid is heavily dependent on the fermentation of sugar and starch derived from crops such as corn and sugarcane [4]. However, the technical development of bio‐based chiral building blocks from lignin is a highly desirable alternative because it does not compete with the globally increasing demand for food. Lignin is the most abundant aromatic compound in nature, making up to 35% of woody biomass. Lignin is mainly utilized as a combustion fuel in pulp and paper manufacturing (≈50 million tons annually), whereas only 1%–2% enter the chemical market as industrial lignin [5, 6]. One of the reasons for the restricted applications of lignin is the heterogeneity of its chemical structure [7, 8]. Therefore, to expand the application scope of lignin and promote the comprehensive utilization of renewable lignocellulosic biomass, the development of technologies to convert lignin into a wide variety of products is essential [9, 10]. Efficient processes to obtain aromatic compounds such as vanillic acid (VA) **1** and protocatechuic acid (PCA) **2** from various types of lignin have been reported [11, 12, 13, 14]. In addition, we previously proposed a technique for combining heterogeneous aromatic compounds obtained from the chemical decomposition of lignin into a single building block molecule, 2‐pyrone‐4,6‐dicarboxylic acid (PDC), using a bacterial convergent metabolism for the first time [15]. This technique has recently attracted attention as a biological funneling strategy [16]. In addition to biodegradable and heat‐resistant bio‐based polymers, strong adhesives for various materials such as metal and glass were also developed using PDC as a building block [17, 18, 19, 20].

3‐Carboxymuconolactone (3CML), a chiral microbial intermediate derived from lignin that exists as a pair of enantiomers, i.e., 4*S*‐3CML **4** and 4*R*‐3CML **5**, can be polymerized in a similar manner to PDC by virtue of its two carboxyl groups. 4*S*‐3CML **4** is produced from the precursor 3‐carboxy‐*cis*,*cis*‐muconate (CMA) **3** by a CMA lactonizing enzyme in the PCA 3,4‐cleavage pathway of fungi such as *Neurospora crassa* and *Aspergillus nidulans* [21, 22, 23]. We recently reported the conversion of lignin‐derived aromatic compounds into optically pure 4*S*‐3CML **4** using an engineered *Pseudomonas putida* strain with plasmids containing bacterial and fungal genes [24]. 4*S*‐3CML **4** is a promising polymer building block for the production of novel materials containing chiral compounds derived from lignin. In fact, we successfully synthesized polyesters and polyurethanes using 4*S*‐3CML **4** as a building block [25].

**SCHEME 1:**
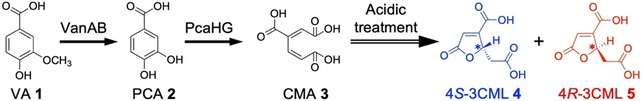
Production pathway of racemic 3CML from VA **1**. The continuous arrows represent pathways mediated by microbial enzymes. The double arrow represents the chemical conversion pathway.

Using the enantiomeric pair 4*S*‐3CML **4** and 4*R*‐3CML **5** as polymer building blocks in appropriate blending ratios can be envisaged as a promising strategy to fabricate novel materials such as polylactic acid with tunable physical properties. However, no microbial metabolic pathways producing 4*R*‐3CML **5** in optically pure form have been reported. Meanwhile, a chemical process for the synthesis of 3CML from CMA **3** via acidic treatment without biological reaction was reported about 70 years ago [26, 27]. The chemically synthesized 3CML was presumed to be in racemic form, although no stereochemical characterization based on enantiomers was reported. Since these pioneering works, there have been no reports on the production of racemic 3CML. The development of a process to produce racemic 3CML from CMA, a lignin‐derived convergent metabolite, is desirable for synthesis of novel materials with blending 4*S*‐3CML **4** and 4*R*‐3CML **5** as polymer building blocks in varying enantiomeric ratios from 100:0 to ≈50:50. In this study, a one‐pot process comprising fed‐batch fermentation and subsequent acidic treatment was developed for the production of racemic 3CML from VA **1**, the major aromatic compound derived from lignin, and racemic 3CML was stereochemically characterized on the basis of the enantiomers (Scheme 1).

## 2 Results and Discussion

### 2.1 Microbial Conversion of VA 1 into CMA 3 and 3CML

**FIGURE 1:**
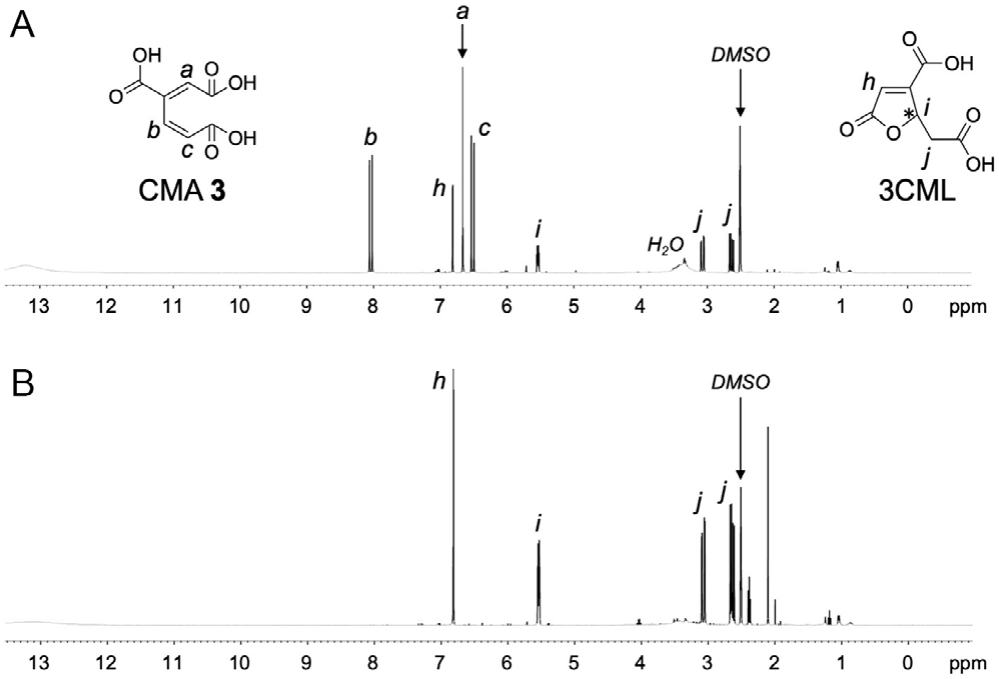
^1^H NMR spectra of (A) VA metabolite mediated by PpY1100/CMA and (B) acid‐treated product of A. Peaks are labeled with alphabetic numbers.

**FIGURE 2:**
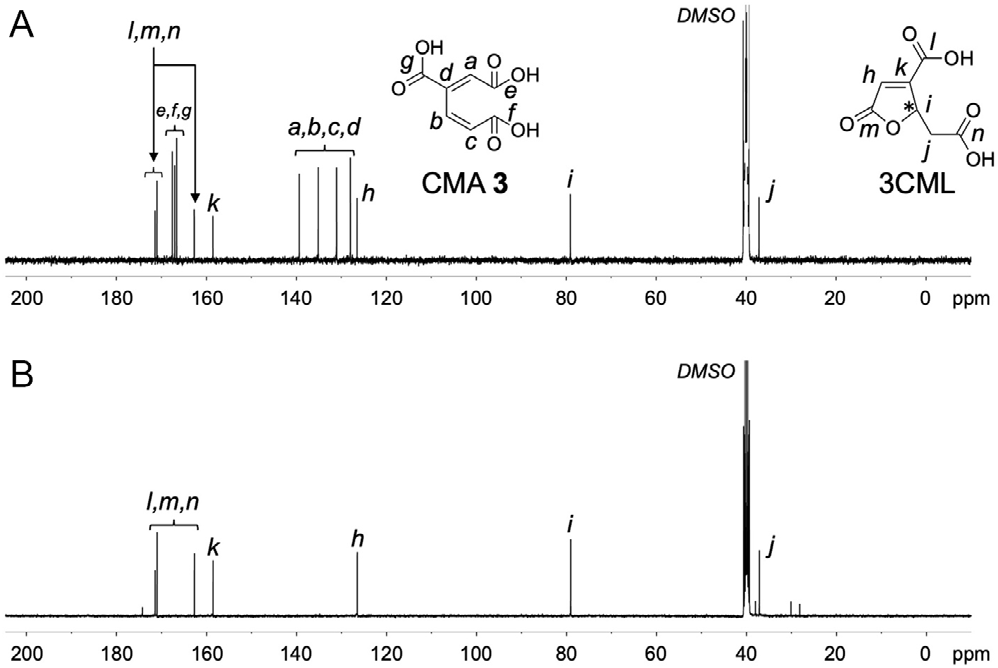
^13^C NMR spectra of (A) VA metabolite mediated by PpY1100/CMA and (B) acid‐treated product of A. Peaks are labeled with alphabetic numbers.

To produce CMA **3** from VA **1**, a bacterial strain *P. putida* PpY1100, whose cells can take up lignin‐derived aromatic compounds such as VA **1** and PCA **2** but cannot metabolize them, was adopted as a host [28]. This host strain was engineered to efficiently convert VA **1** into CMA **3** by introducing the plasmid pKCMA02, which carries the VA **1** demethylase gene (*vanAB*) and PCA 3,4‐dioxygenase gene (*pcaHG*) of *P. putida* KT2440 (Scheme 1). The resulting strain was termed PpY1100/CMA. To make the productive ability of CMA **3** independent of the induction, these genes were transcribed from *lac* promoters and highly expressed in the host strain. A batch test was performed to examine the ability of PpY1100/CMA to produce CMA **3** from VA **1**. Specifically, PpY1100/CMA was cultured in the presence of 5 mM VA **1** at 28°C, and the CMA **3** production was evaluated by combining UV–visible absorption spectroscopy, thin‐layer chromatography (TLC), high performance liquid chromatography (HPLC), and NMR analyses owing to its predicted chemical instability according to previous reports [26, 27]. At 24 h after inoculation, VA **1** and the PCA **2** intermediate were completely metabolized (Figure S1), the UV–visible spectrum of the ethyl acetate extract of the culture medium showed an absorption peak specific to CMA **3** at around 263 nm (Figure S2) [27]. The TLC analysis of an ethyl acetate extract showed two products (Product I and Product II), among which Product II exhibited a retention factor that was consistent with that of authentic 4*S*‐3CML **4** (Figure S3). To determine the chemical structures of the two products, the ethyl acetate extract of the culture medium was analyzed via ^1^H and ^13^C NMR spectroscopy. The ^1^H NMR (400 MHz, [D_6_]DMSO, 25°C) spectrum showed the major signals at δ 6.51 (1H, d, *J* = 16.1 Hz), 6.66 (1H, s), and 8.03 (1H, d, *J* = 16.1 Hz) ppm, and the main resonances in the ^13^C NMR (100 MHz, [D_6_]DMSO, 25°C) spectrum appeared at δ 127.5, 130.6, 134.7, 139.0, 166.1, 166.6, and 167.1 ppm, which agreed with those estimated for CMA **3** (Figures 1A and 2A). In addition, minor signals appeared at δ 2.64 (1H, dd, *J* = 16.5, 8.0 Hz), 3.07 (1H, dd, *J* = 16.6, 3.2 Hz), 5.53 (1H, ddd, *J* = 8.0, 3.2, 2.1 Hz), and 6.81 (1H, d, *J* = 2.1 Hz) ppm in the ^1^H NMR spectrum and at δ 36.7, 78.6, 126.0, 158.1, 162.2, 170.5, and 171.0 ppm in the ^13^C NMR spectrum, which corresponded to those of authentic 4*S*‐3CML **4** (Figures S4A and S5A). These results indicate that VA **1** was converted into CMA **3**, which in turn was partially converted into 3CML.

### 2.2 Chemical Conversion of CMA 3 into Racemic 3CML via Acidic Treatment

4*S*‐3CML **4** is an intermediate in the PCA **2** metabolic pathways of fungi such as *Neurospora crassa* and *Aspergillus nidulans* and is not produced by a bacterial host (Scheme 1) [21, 22, 23]. To confirm that the 3CML found in the culture medium was chemically produced from CMA **3** rather than biologically produced by PpY1100/CMA, acidic treatment was performed. The culture medium resulting from VA **1** was acidified to pH 2.0 by adding a phosphoric acid solution and incubated at 28°C after the bacterial cells were removed. The UV–visible spectrum of the ethyl acetate extract of the reaction mixture at 48 h after the acidification showed an absorption peak specific to 3CML at around 213 nm (Figure S2). The ^1^H and ^13^C NMR spectra only showed signals specific to 3CML and none for CMA **3** (Figures 1B and 2B), indicating that 3CML contained in the culture medium was chemically produced from CMA **3**.

**FIGURE 3:**
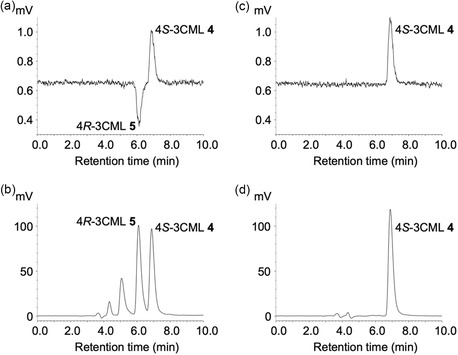
Chiral HPLC–CD analyses of 3CML enantiomers. The acid‐treated product (a,b) and authentic 4*S*‐3CML **4** (c,d) were separated on a chiral HPLC column and detected using CD (a,c) and UV (b,d) detectors.

A chiral HPLC–circular dichroism (CD) system was employed to confirm whether the generated 3CML was in a racemic form. The product of the acidic treatment was found to contain approximately equal molar amounts of two enantiomers, which eluted at retention times of 6.1 and 6.9 min, according to the peak areas (Figure 3a,b). The enantiomer eluted at 6.9 min corresponded to authentic 4*S*‐3CML **4** (Figure 3c,d); therefore, the enantiomer eluted at 6.1 min was identified as 4*R*‐3CML **5**. This stereochemical characterization based on enantiomers confirmed that 3CML was generated in a racemic form.

### 2.3 Effect of pH on Racemic 3CML Production

**FIGURE 4:**
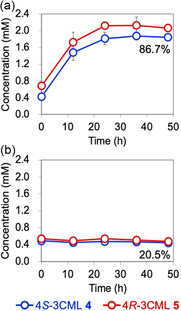
(a) Racemic 3CML production via acidic treatment at pH 2.0. (b) The control reaction mixture at pH 6.5. The 3CML enantiomers at 0 h were derived from those produced during microbial conversion mediated by PpY1100/CMA. The resulting culture media from VA 1 (5 mM) were diluted 0.9 times by adding a phosphoric acid solution for pH adjustment (see Experimental Section). The molar yields at 48 h after acidification were calculated by comparing the molar quantities between VA 1 and a 3CML enantiomer (shown in the graphs). The error bars indicate the standard deviation of triplicate experiments.

The effect of pH on the production of racemic 3CML was quantitatively analyzed using a chiral HPLC–CD system. The concentrations of 4*S*‐3CML **4** and 4*R*‐3CML **5** increased in the conversion reaction at pH 2.0, reaching 1.84 and 2.06 mM with a molar yield of 86.7% as a racemic form at 48 h after acidification, respectively (Figure 4a). In contrast, in the conversion reaction at pH 6.5, which was performed as a control, the respective concentrations only reached 0.44 and 0.48 mM with a molar yield of 20.5% as a racemic form (Figure 4b). Thus, according to this quantitative characterization based on the enantiomers, the chemical conversion of CMA **3** into racemic 3CML depended on pH and was promoted under acidic conditions.

### 2.4 One‐Pot Process for Racemic 3CML Production Using a Jar Fermenter

**FIGURE 5:**
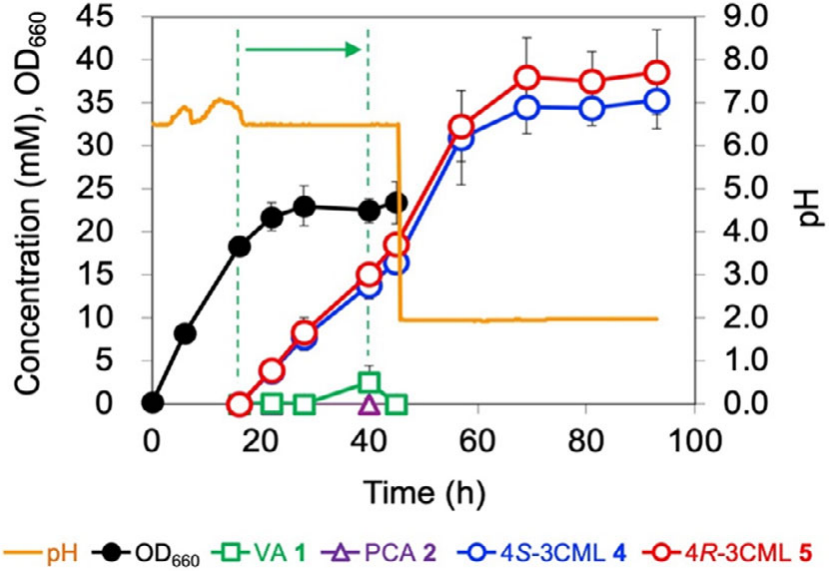
Racemic 3CML production mediated by PpY1100/CMA and subsequent acidic treatment using a jar fermenter. The arrow indicates the time‐point of VA 1 feeding. The error bars indicate the standard deviation of triplicate experiments.

To establish a method to produce racemic 3CML at a larger scale, a one‐pot process combining fed‐batch fermentation with subsequent acidic treatment was conducted using a jar fermenter with an initial culture volume of 250 mL. PpY1100/CMA was grown at 28°C until an optical density at 660 nm (OD_660_) of ≈20 was obtained, and 17.8 mmol (3 g) VA 1 was then continuously fed into the fermenter for ≈24 h. The production of 3CML enantiomers was monitored from the start of VA 1 feeding because they were also produced during microbial conversion under neutral pH conditions (see ‘Microbial conversion of VA 1 into CMA 3 and 3CML’ section). At the end of VA 1 feeding, 2.5 mM VA 1 accumulated in the culture medium. Therefore, only the glucose solution was additionally fed for 5 h, resulting in the complete conversion of the accumulated VA 1 (Figure 5).

After fed‐batch fermentation, acidic treatment was performed at 28°C by adding a phosphoric acid solution into the fermenter using a peristaltic pump to adjust the pH to 2.0. The concentrations of 4*S*‐3CML **4** and 4*R*‐3 ML **5** increased, reaching 35.3 mM (6.6 g/L) and 38.6 mM (7.2 g/L) with an enantiomeric ratio of 48:52 and a molar yield of 93.1% as a racemic form at 48 h after acidification, respectively (Figure 5). These values are highly comparable to those in our previous report for enantioselective 4*S*‐3CML **4** production from lignin derivatives [24]. These results confirm that the one‐pot process combining fed‐batch fermentation with subsequent acidic treatment using a jar fermenter enabled the large‐scale production of racemic 3CML from VA **1**.

Racemic 3CML was isolated in a yield of 45.5% (average of triplicate experiments) from the solution after acidic treatment via simple solvent extraction and recrystallization using ethyl acetate, which is considered as a green solvent owing to its low environmental impact [29]. No other substances such as CMA **3** were detected (Figures S4B and S5B). Although not performed in this work, repeating the recrystallization using a filtrate and recovering and recycling the ethyl acetate solvent via evaporation could further reduce the purification costs.

## 3 Conclusion

This study demonstrates that 3CML is a novel chiral building block that can be produced enantioselectively or nonenantioselectively at will from lignin derivatives. Racemic 3CML was produced from VA **1**, the major aromatic compound derived from lignin, for the first time. The engineered strain could efficiently convert VA **1** into CMA **3**, a precursor of racemic 3CML. Then, the pH‐dependent chemical conversion of CMA **3** into racemic 3CML was promoted under acidic conditions. Furthermore, a one‐pot process for the production of racemic 3CML at a larger scale was established by combining fed‐batch fermentation with subsequent acidic treatment, allowing the use of a reduced volume of solvent. The developed process consistently proceeds at 28°C and does not require pressure or metal reagents, which is advantageous for industrial applications. The product was stereochemically identified as a racemic form on the basis of its enantiomers for the first time. This strategy could be extended to other aromatic compounds obtained from industrial lignin [11, 12, 13, 14] by applying the bacterial convergent metabolism [30]. By combining the nonenantioselective process developed in this study with the enantioselective process yielding an optically pure enantiomer [24], blend polymers of 4*S*‐3CML **4** and 4*R*‐3CML **5** with varying enantiomeric ratios from 100:0 to 48:52 could be generated. At this stage, the synthesis of blend polymers with a higher content of 4*R*‐3CML **5** would require separating this enantiomer from the racemic form using chiral HPLC. Future work should focus on the separation or production of 4*R*‐3CML **5** in optically pure form, which could be achieved by (1) optically resolving the racemic form through the conversion of only 4*S*‐3CML **4** into the other substance using a fungal stereoselective metabolism, (2) identifying microorganisms and enzymes that stereoselectively produce 4*R*‐3CML **5** from nature, and (3) artificially modifying the stereoselectivity of enzymes using a genetic engineering technique.

## 4 Experimental Section

### 4.1 Chemicals

An authentic 4*S*‐3CML **4** sample of known optical purity was used [24]. 4*R*‐3CML **5** was collected from recrystallized racemic 3CML using a HPLC system (JASCO Corp.) equipped with a CHIRAL ID column (5 μm column, 10 × 250 mm; Daicel Corp.) and a UV970 detector (JASCO Corp.) set to 240 nm. The collection was performed with an isocratic mobile phase of hexane (80.0%), ethanol (20.0%), and trifluoroacetic acid (0.1%), at 40°C and a flow rate of 1.4 mL/min. The collected substance was rotary evaporated to remove the solvent and dried up completely in a vacuum oven after washing three times with chloroform. The resulting authentic 4*R*‐3CML **5** was used after confirming its optical purity. Bacto tryptone and yeast extract were purchased from Thermo Fisher Scientific Inc., and other reagents were purchased from Wako Pure Chemical Industries, Ltd. and Tokyo Chemical Industry Co., Ltd.

### 4.2 Microbial Conversion of VA 1 into CMA 3

To efficiently convert VA **1** into CMA **3**, the *vanAB* (*PP*_3736–3737) and *pcaHG* (*PP*_4656–4655) genes of *P. putida* KT2440 (ATCC 47 054) were cloned into pKT230MC [24], and the resulting plasmid was named pKCMA02 (Scheme 1). pKCMA02 was introduced into *P. putida* PpY1100, which was named PpY1100/CMA. The *vanAB* and *pcaHG* genes were transcribed from the *lac* promoters and highly expressed in PpY1100/CMA without an inducer. PpY1100/CMA was inoculated into a shaking flask with baffles containing 100 mL culture medium composed of 10 g/L Bacto tryptone, 5 g/L yeast extract, 5 g/L NH_4_Cl, and 25 mg/L kanamycin and incubated at 28°C and 160 rpm for 24 h. The culture growth was determined by monitoring the OD_660_ using a spectrophotometer (V‐630BIO; JASCO Corp.). The resulting cells were harvested via centrifugation and resuspended in 10 mL of the same culture medium. The cell suspension was inoculated into a shaking flask with baffles containing 500 mL of the same culture medium and 5 mM VA **1** with an initial OD_660_ of 0.2 and incubated at 28°C and 160 rpm. Batch tests were performed in triplicate. Samples (5 mL) were periodically taken and analyzed for intermediates and culture growth (OD_660_). A sample was acidified to less than pH 2.0 using a HCl solution and quickly extracted with an equal amount of ethyl acetate when necessary. The solvent in the ethyl acetate extract was removed via rotary evaporation.

### 4.3 Chemical Conversion of CMA 3 into Racemic 3CML

The resulting bacterial cells were removed via centrifugation and filtration through a membrane with a porosity of 0.2 µm. The supernatant solution (135 mL) was adjusted to pH 2.0 by adding a phosphoric acid solution, and the volume was then adjusted to 150 mL by adding ultrapure water. This solution was put into a flask with a screw cap and stirrer and incubated at 28°C and 300 rpm. A reaction test with the pH adjusted to 6.5 was also performed as a control. The acidic treatment tests were performed in triplicate. Samples (5 mL) were periodically taken and extracted with an equal amount of ethyl acetate. The solvent in the ethyl acetate extract was removed via rotary evaporation, and the 3CML enantiomer content was then analyzed. The molar yields (%) were calculated by comparing the molar quantities between VA **1** and the 3CML enantiomer.

### 4.4 Racemic 3CML Production Using a Jar Fermenter

PpY1100/CMA was inoculated into an L‐shaped test tube containing the same culture medium (10 mL) as described and incubated at 28°C and 160 rpm for 24 h. The cultured strain (1 mL) was inoculated again into a shaking flask with baffles containing the same culture medium (100 mL) and then incubated at 28°C and 160 rpm for 8 h. The resulting strain was centrifuged and resuspended in 10 mL of the same culture medium. The strain suspension was inoculated into a jar fermenter (BMS‐10NP3; ABLE Corp.) containing 250 mL of final culture medium of the same composition as above with an initial OD_660_ of 0.2. The final growth culture was supplemented by feeding a 588 mg/mL glucose solution at a flow rate of 0.408 mL/h (240 mg/h glucose) using a peristaltic pump. The bioreactor was maintained at 28°C and an aeration of 3.5 L/min with agitation at 700 rpm while controlling the pH at 6.5‐7.1 with 1.48 M ammonia solution and 0.67 M phosphoric acid solution. When excessive bubbles were generated, Adekanol LG‐109 (ADEKA Corp.) was added as a defoamer. When the culture growth (OD_660_) reached ≈20, the feed of a single glucose solution was stopped and replaced with a feedstock solution (30 mL; pH 8.2) composed of 3 g VA **1** and 6 g glucose at a flow rate of 1.2 mL/h (120 mg/h VA **1** and 240 mg/h glucose). After fed‐batch fermentation, an acidic treatment was performed at 28°C by adding ≈5 mL of 14.6 M phosphoric acid solution to the fermenter using a peristaltic pump to adjust the pH to 2.0. Aeration was stopped with agitation at 100 rpm. The jar fermenter tests were performed in triplicate. Samples (5 mL) were periodically taken and analyzed for intermediates, products, and culture growth (OD_660_). The values of the titers (g/L) of 3CML enantiomers correspond to the concentration at the end of the acidic treatment.

### 4.5 Recrystallization

The resulting reaction mixture (20 mL) was extracted three times with an equal amount of ethyl acetate. The water dissolved in the ethyl acetate extract was removed using sodium sulfate anhydrate, and the solution was then rotary evaporated and dried completely using a vacuum oven. The dried extract was redissolved in 10 mL of dehydrated ethyl acetate while heating at 60°C, and the solution was then rotary evaporated to ≈5 mL and kept overnight at 4°C. The precipitates were collected by filtration and then completely redried using a vacuum oven.

### 4.6 Analytical Methods

To determine the 3CML enantiomer content, each extract was analyzed using an HPLC system (JASCO Corp.) equipped with a CHIRAL ID column (5 μm column, 4.6 × 250 mm; Daicel Corp.) and a CD‐1595 detector (JASCO Corp.) set to 240 nm. The analyses were performed with an isocratic mobile phase of hexane (80.0%), ethanol (20.0%), and trifluoroacetic acid (0.1%), at 40°C and a flow rate of 1 mL/min. The UV–visible spectrum of each extract dissolved in water was analyzed by scanning the UV spectrum from 300 to 200 nm using a spectrophotometer (V‐630BIO; JASCO Corp.) at a scan rate of 400 nm/min. Ethyl acetate extracts from the culture media were separated via TLC on a silica gel 60 F254 plate (Merck KGaA) using the developing solvent, chloroform, ethyl acetate, and formic acid (10:8:1, v/v/v). Spots on TLC were visualized by UV light. ^1^H and ^13^C NMR spectra were measured with a BRUKER AVANCE III HD 400 MHz spectrometer (Bruker Corp.). VA **1** and PCA **2** in a culture medium were analyzed as described previously [24].

## Supporting Information

Additional supporting information can be found online in the Supporting Information section. **Supporting** **Fig.** **S1**: Microbial conversion for VA **1** metabolism mediated by PpY1100/CMA. The error bars indicate the standard deviation of triplicate experiments. **Supporting** **Fig.** **S2**: UV–visible spectra of VA **1** metabolite mediated by PpY1100/CMA (green), acid‐treated product (red), and authentic 4*S*‐3CML **4** (blue). **Supporting** **Fig.** **S3**: TLC analysis of microbial conversion for VA **1** metabolism mediated by PpY1100/CMA. Lanes 1, 2, and 3 indicate authentic VA **1**, PCA **2**, and 4*S*‐3CML **4**, respectively; lanes 4–8 correspond to microbial conversion for 0, 6, 12, 18, and 24 h, respectively. **Supporting** **Fig.** **S4**: ^1^H NMR spectra of (**A**) authentic 4*S*‐3CML **4**, and (**B**) recrystallized racemic 3CML. Peaks are labeled with alphabetic numbers. **Supporting** **Fig.** **S5**: ^13^C NMR spectra of (**A**) authentic 4*S*‐3CML **4**, and (**B**) recrystallized racemic 3CML. Peaks are labeled with alphabetic numbers.

## Author Contributions

**Yuzo Suzuki**: conceptualization (lead), data curation (lead), formal analysis (lead), funding acquisition (lead), investigation (lead), methodology (lead), project administration (lead), resources (lead), supervision (lead), validation (lead), visualization (lead), writing – original draft (lead), writing – review & editing (lead). **Takuma Araki**: funding acquisition (supporting), writing – review & editing (supporting). **Masaya Fujita**: funding acquisition (supporting), writing – review & editing (supporting). **Naofumi Kamimura**: funding acquisition (supporting), writing – review & editing (supporting). **Eiji Masai**: funding acquisition (lead), writing – review & editing (supporting). **Tsuyoshi Michinobu**: funding acquisition (supporting), methodology (equal), writing – review & editing (supporting). **Yuichiro Otsuka**: resources (equal), writing – review & editing (supporting). **Shojiro Hishiyama**: investigation (equal), writing – review & editing (supporting). **Masaya Nakamura**: conceptualization (lead), funding acquisition (supporting), resources (equal), writing – review & editing (supporting).

## Funding

This work was supported by the Japan Society for the Promotion of Science (22K05772); Japan Science and Technology Agency (JPMJPF2104).

## Conflicts of Interest

The authors declare no conflicts of interest.

## Supporting information

Supplementary Material

## Data Availability

The data that support the findings of this study are available from the corresponding author upon reasonable request.
