# Supplementary material for: Sustainable Nonenantioselective Production and Stereochemical Characterization of the Lignin‐Derived Chiral Building Block 3‐Carboxymuconolactone
Source: ChemistryOpen. 2026 Jan 5;15(1):e202500453. doi: 10.1002/open.202500453 (PMC12771582; doi:10.1002/open.202500453)
Supplement: Supplementary file 1 — Supplementary Material [file OPEN-15-e202500453-s001.pdf]

Supporting Information

Sustainable Nonenantioselective Production and Stereochemical Characterization of the Lignin-Derived Chiral Building Block 3-Carboxymuconolactone

Yuzo Suzuki,*[a] Takuma Araki,[a] Masaya Fujita,[b] Naofumi Kamimura,[b] Eiji Masai,[b] Tsuyoshi Michinobu,[c] Yuichiro Otsuka,[a] Shojiro Hishiyama,[a] Masaya Nakamura[a]

[a] *Department of Forest Resource Chemistry, Forestry and Forest Products Research Institute, Tsukuba, Ibaraki 305-8687 Japan*

[b] *Department of Materials Science and Bioengineering, Nagaoka University of Technology, Nagaoka, Niigata 940-2188, Japan*

[c] *Department of Materials Science and Engineering, Institute of Science Tokyo, Meguro-ku, Tokyo 152-8552, Japan*

**Yuzo Suzuki, E-mail: suzuki_yuzo710@ffpri.go.jp*

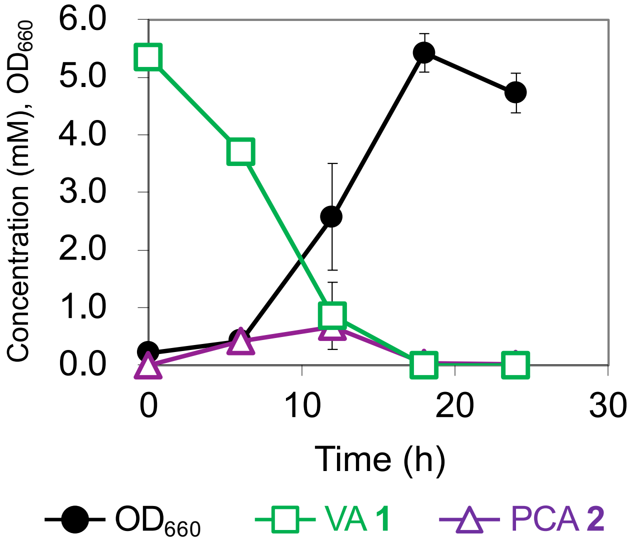

Figure S1. Microbial conversion for VA **1** metabolism mediated by PpY1100/CMA. The error bars indicate the standard deviation of triplicate experiments.

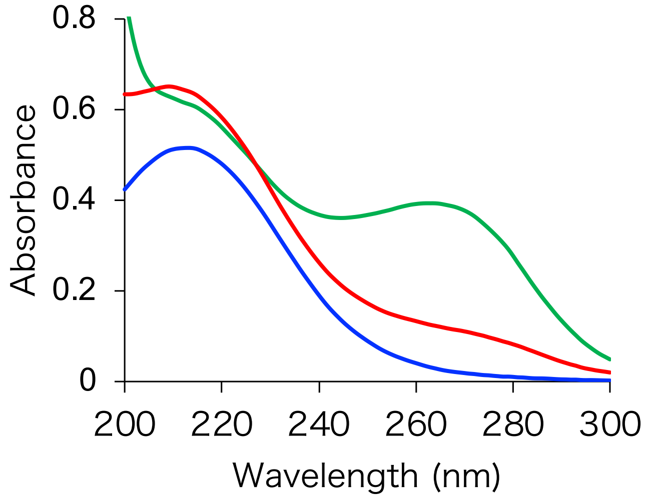

Figure S2. UV–visible spectra of VA **1** metabolite mediated by PpY1100/CMA (green), acid-treated product (red), and authentic 4*S*-3CML **4** (blue).

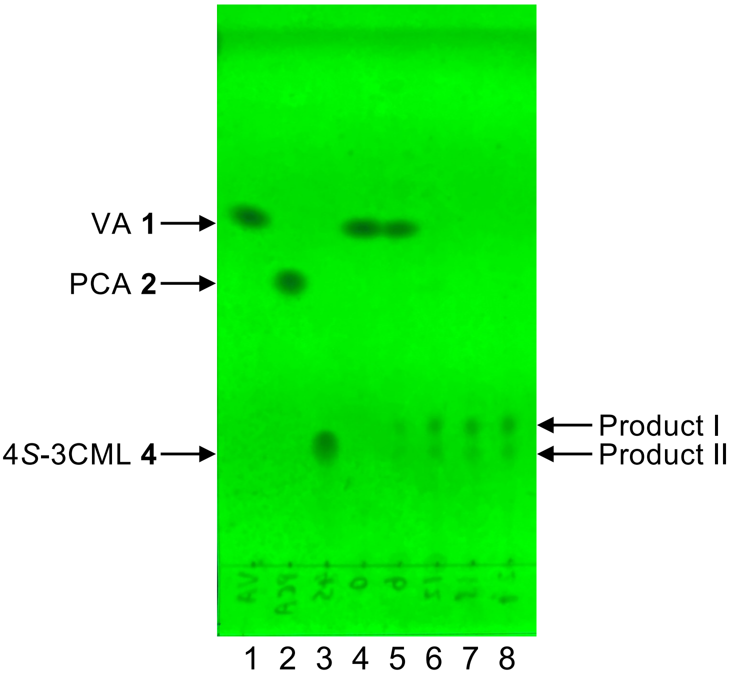

Figure S3. TLC analysis of microbial conversion for VA **1** metabolism mediated by PpY1100/CMA. Lanes 1, 2, and 3 indicate authentic VA **1**, PCA **2**, and 4*S*-3CML **4**, respectively; lanes 4–8 correspond to microbial conversion for 0, 6, 12, 18, and 24 h, respectively.

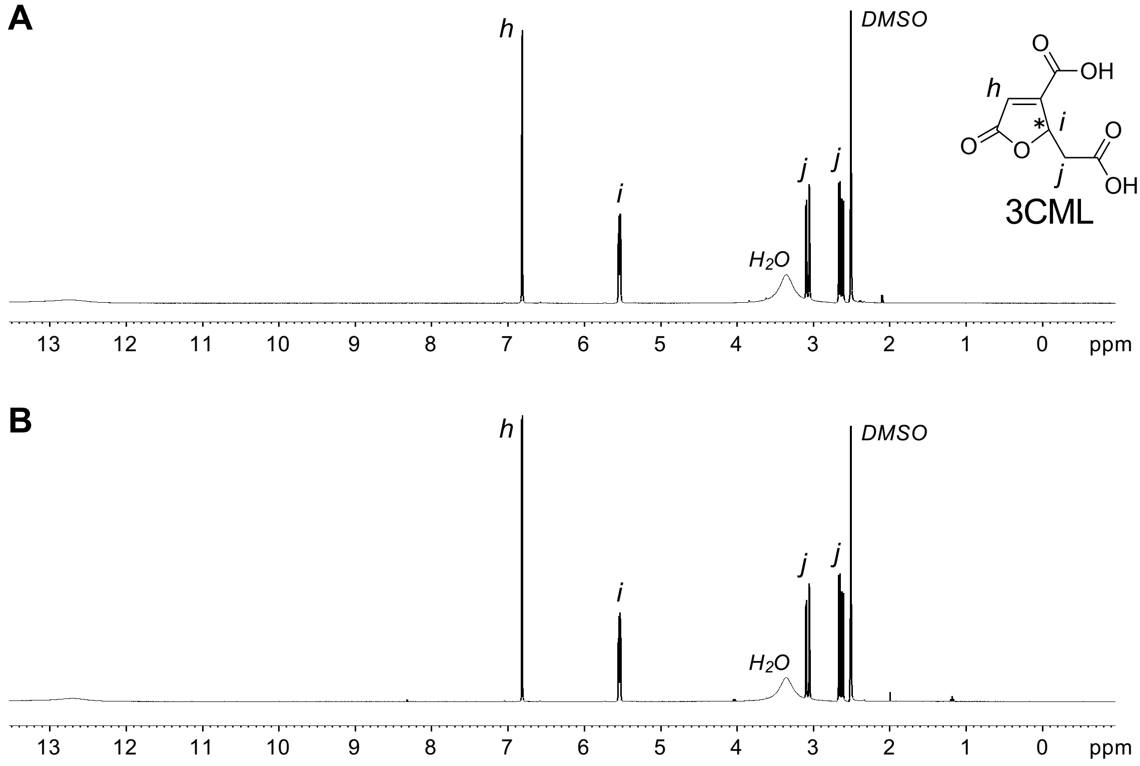

Figure S4. ^{1}H NMR spectra of (**A**) authentic 4*S*-3CML **4**, and (**B**) recrystallized racemic 3CML. Peaks are labeled with alphabetic numbers.

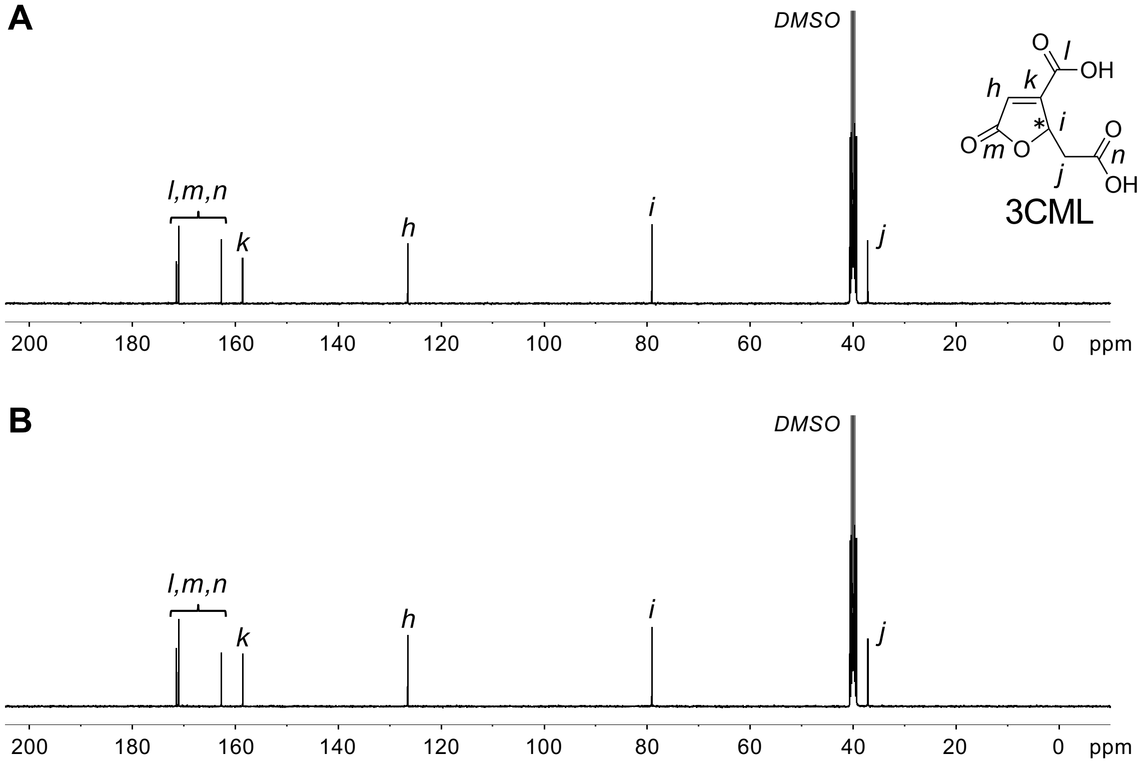

Figure S5. ^{13}C NMR spectra of (**A**) authentic 4*S*-3CML **4**, and (**B**) recrystallized racemic 3CML. Peaks are labeled with alphabetic numbers.
